# Sentiment Analysis of European Bonds 2016–2018

**DOI:** 10.3389/frai.2019.00020

**Published:** 2019-10-15

**Authors:** Peter Schwendner, Martin Schüle, Martin Hillebrand

**Affiliations:** ^1^School of Management and Law, Center for Asset Management, Zurich University of Applied Sciences, Winterthur, Switzerland; ^2^School of Life Sciences and Facility Management, Institute for Applied Simulation, Zurich University of Applied Sciences, Wädenswil, Switzerland; ^3^European Stability Mechanism, Luxembourg, Luxembourg

**Keywords:** sovereign bonds, contagion, sentiment, European sovereign bond crisis, correlation, correlation influence, networks

## Abstract

We revisit the discussion of market sentiment in European sovereign bonds using a correlation analysis toolkit based on influence networks and hierarchical clustering. We focus on three case studies of political interest. In the case of the 2016 Brexit referendum, the market showed negative correlations between core and periphery only in the week before the referendum. Before the French presidential elections in 2017, the French bond spread widened together with the estimated Le Pen election probability, but the position of French bonds in the correlation blocks did not weaken. In summer 2018, during the budget negotiations within the new Italian coalition, the Italian bonds reacted very sensitively to changing political messages but did not show contagion risk to Spain or Portugal for several months. The situation changed during the week from October 22 to 26, as a spillover pattern of negative sentiment also to the other peripheral countries emerged.

## Introduction

In this empirical study, we discuss the short-term impact of three specific political situations relevant to the European Union on the return correlations between its sovereign bond markets in 2016, 2017, and 2018. We focus on effects happening at the same time in these markets and interpret the correlation patterns on an hourly timescale in non-overlapping weekly time windows as an expression of the sentiment of market makers regarding a potential risk spillover. Forbes and Rigobon (2002) and Rigobón (2019) present a precise differentiation of “spillover,” “contagion,” and “interdependence” phenomena.

To illustrate our interpretation of “sentiment,” we point out that positioning decisions of large investors happen at a slower pace than quote changes generated by quote machines of bond market makers. Quote machines need to make sure that market makers cannot get “arbitraged” by external traders who have access to all public market information. Therefore, market maker quotes need to include current market information, even information inferred from other markets. These “cross-sectional” quotation models can enable correlation patterns in the quoted time series. For example, negative news concerning a specific country may trigger a spread widening of bonds of this country and also of bonds of other, similar countries even before many actual trades happen. The changes in observed quotes then may have an impact on the trading decisions of speculative traders who might follow a momentum trading rule. On a longer time scale, these quote changes can also have an impact on the positioning of long-term investors who might be forced to cut their positions as the need to comply with a stop-loss or value-at-risk rule.

The Euro-denominated sovereign bond markets within the European Union are a very specific universe, as the yield levels across countries significantly converged already before the introduction of the Euro in 1999 and diverged during the European sovereign debt crisis from 2010 to 2014, accompanied by a pronounced block structure in the correlation matrix reflecting the “core-periphery” dichotomy. At the peak of the crisis between 2010 and 2012, the correlations between core European and periphery bonds have even been negative as only the core bonds acted as “safe havens,” but not the periphery bonds, inducing capital flows from the weaker to the stronger bond markets.

The spread increase in Euro area bonds from 2010 to 2012 has been discussed thoroughly by academia as well as by central bank research and related European institutions, for example by Beirne and Fratscher (2013) and Tola and Waelti (2015). D'Agostino and Ehrmann (2014) pointed to an overreaction of the market given the change in fundamentals and thus to a structural change in longer-term risk perception. Gross and Kok (2013); Alter and Beyer (2014); Broner et al. (2014); Glover and Richards-Shubik (2014); Shoesmith (2014); Erce (2015); Li and Waterworth (2016); Lange et al. (2017) discussed the relationships between private and public sector bonds, between sovereign bonds and credit derivatives, and the transmission channels between bank risk and sovereign risk. Gerlach-Kristen (2015); Blasques et al. (2016); Ehrmann and Fratzscher (2017); Moessner (2018); Arakelian et al. (2019) confirmed the stabilizing impact of ECB measures on bond spreads after 2012.

Many of these authors use variations of the Diebold and Yilmaz (2014) variance-decomposition framework that allows applying network theory to interpret the time-lagged variance contributions as variance spillover effect between markets.

Schwendner et al. (2015) applied a correlation influence approach from Kenett et al. (2010). This approach does not employ a time lag structure and therefore, does not address realized variance spillover across time, but the current perception regarding spillover risk reflected in bond correlations. In contrast to correlations, the concept of correlation influence is a directed measure from a market *A* to another market *B* that explains correlations between market *B* and all other markets. A noise filter using a bootstrap scheme allows dropping the less significant correlation influences and thus to identify the markets that have the highest explanatory power regarding the correlation matrix. The authors found positive correlations dominating the European bond markets from 2004 to 2009. Between 2010 and 2012, negative correlations between the core and periphery markets had the highest explanatory power for the European bond market correlations. The situation normalized in 2013 and 2014, but negative correlations between core and periphery and negative correlation influences reappeared during the negotiations between Greece and the Eurogroup in the first half of 2015. Contagion risk and a possible breakup of the Euro area was no more an abstract risk but even used as negotiation leverage.

After the agreement to the third ESM-funded Euro area financial assistance program in July 2015, bond spreads and contagion risk declined substantially. Media focus switched to the increasing influx of refugees from Syria, Afghanistan, Iraq, and African countries to Europe that peaked in October 2015 and a wave of terrorist attacks after that. Populist parties gained substantially since then by stressing anti-immigration positions even more than anti-austerity and anti-EU postures.

Before the Brexit referendum on June 23, 2016, most studies warned of the negative economic consequences of a potential Brexit (Boettcher, 2016; EIU, 2016; Kierzenkowski et al., 2016). The unexpected Brexit outcome was explained afterwards by immigration fears and distrust in established media being more convincing than abstract rational economic arguments. The impact on bond markets was small as a decline of the British pound relative to the Euro absorbed the Brexit shock.

In the Dutch general elections on March 15, 2017, the right-wing PVV gained grounds, but finally, a four-party conservative-social-liberal coalition formed a new government in October 2017. During the presidential elections in France in spring 2017, the most important topics were the relationship toward the EU and immigration. The spread between French and German bonds closely followed the odds of the right-wing Marie Le Pen winning in the second round (Bird and Sindreu, 2017; Macintosh, 2017). After Emmanuel Macron won the second round on May 7, 2017, Europe embraced a wave of positive mood, and sovereign spreads declined (Whittall, 2017). The next risk scenario highlighted by the financial press (Marriage and Jennifer, 2017) was a Eurosceptic government in Italy after the next elections and a potential exit from the EU (Kelly et al., 2015).

The Italian general elections on March 4, 2018, indeed resulted in gains for the populist five stars movement and the right-wing Lega, but not immediately in a new government or a sharp reaction of financial markets. Sandhu (2018) noted a large demand for Euro-denominated sovereign bonds from Asian investors who have a very low funding rate. The BTP-Bund spread widened and whipsawed during the formation phase of the new government until the end of May. Giuseppe Conte took office as a new prime minister on June 1st and confirmed increased spending commitments. During July and August, the spread lowered slightly. Italian bonds showed increasing volatility as the negotiations for the 2019 budget proceeded (O'Brien, 2018) and both parties postured against the Maastricht criteria. However, in contrast to the 2015 situation with Greece, the spillover to other peripheral countries in the form of increasing Bund spreads was limited (Macintosh, 2018), despite the larger size of the Italian economy and bond market. The limited spillover was reasoned with increasing economic resilience in those countries (Pascual et al., 2018) and contrasted a 2015 “Eurozone Meltdown” risk scenario developed by Kelly et al. (2015).

## Data and Methods

The 10y bonds are the most liquid “benchmark bonds” in the sovereign bond market. For the larger European bond markets (UK, Germany, France, Italy, Spain, and Switzerland), the ICE and EUREX derivatives exchanges offer bond futures as a risk management, hedging and speculation instrument. The open interest of bond futures is much lower than the outstanding volume of bond issues, but bond futures trade at lower bid-ask spreads than bonds and don't require full funding of their market value, so they are the preferred tool for fast intraday trading. EUREX introduced the Spanish BONO bond futures as recently as 2015 as the Spanish bond yields deviated from the Italian bond yields that were previously often used as a proxy for Spanish sovereign risk (EUREX, 2018). Bond market makers often link their bond quotes to the higher-frequency bond futures market to capture short-term market movements in their bond quotes (Allen, 2018; Stafford and Allen, 2018). Therefore, the trading of bond futures instruments can have an impact on the quotes of the much larger bond market.

**Figure 1:**
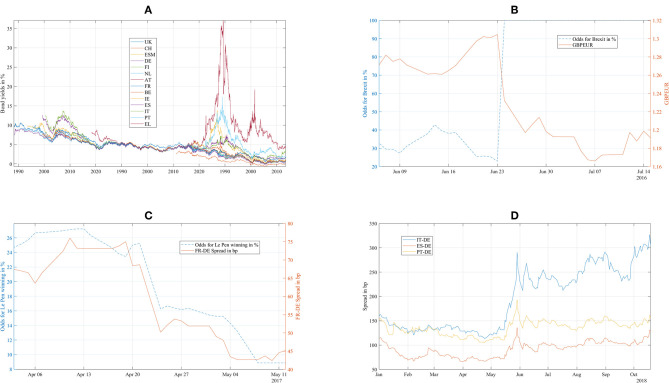
**(A)** European sovereign bond yields from January 2015 to October 2018. **(B)** Brexit odds as estimated by Oddschecker (lhs) and GBPEUR exchange rate (rhs). **(C)** Odds of Le Pen winning as estimated by Oddschecker (lhs) and FR-DE Bond Spread (rhs). **(D)** Spreads of Italian (IT), Spanish (ES), and Portuguese (PT) bonds against Germany (DE).

For this paper, we use a dataset of hourly generic 10y bond yields (Figure 1) from Bloomberg for UK, Switzerland (CH), ESM, Germany (DE), Finland (FI), the Netherlands (NL), Austria (AT), France (FR), Belgium (BE), Ireland (IE), Spain (ES), Italy (IT), Portugal (PT), and Greece (EL). In contrast to our 2015 paper, we added the UK to discuss the Brexit impact and Switzerland to have another non-EUR denominated reference beyond the UK. To get intraday ESM bond yields, we use the current 10y ESM benchmark price quote and compute the yields from those.

From the proprietary EFSF/ESM primary and secondary market databases (source: ESM, 2018), we got insight into the net flows of specific investor types into EFSF and ESM bonds (Supplementary Table 3) to investigate if risk-on/off signals that we see in the correlation patterns have corresponding flow patterns in the trade data. The flows from Asian investors are especially interesting to get an external view on the risk and reward perception of the Euro area, even though FX dynamics may add some noise on the data. Two mechanisms let risk-reward perception having an impact on secondary market flows: the first mechanism is a so-called “flight-to-safety” reaction that lets investors shift bond positions within the Euro area, into the safe assets. For EFSF/ESM bonds, this means net bond inflows. The second mechanism is the reaction to the decision to reduce exposure to the Euro area bond market as a whole. For EFSF/ESM bonds, this means net bond outflows. These mechanisms may happen at the same time and then partially neutralize each other, meaning that some investors are shifting EUR bond exposure to EFSF/ESM and some investors are reducing their overall EUR bond exposure, including EFSF/ESM.

The three political situations in Europe relevant for bond markets that gained the most public interest after 2015 were the 2016 Brexit referendum, the 2017 French presidential elections, and the 2018 Italian budget negotiations. For a detailed quantitative analysis, we picked a time window of 6 weeks for each of these three situations:

- 2016 Brexit referendum: June 6, 2016, to July 15, 2016. The actual day of the referendum was on June 23, 2016.
- 2017 French presidential elections: April 3, 2017, to May 12, 2017. The first round of the elections took place on April 23, the second round on May 7.
- 2018 Italian budget negotiations: September 17, 2018, to October 26, 2018. The deadline to submit the Italian budget to the EU commission was October 15.

Following Schwendner et al. (2015), we use the Pearson correlation coefficient $C_{ij}=\frac{<r_{i}r_{j}>-<r_{i}><r_{j}>}{\sigma_{i}\sigma_{j}}$ of the bond return time series $r_{i}^{t}$ and $r_{j}^{t}$ between two markets *i* and *j* for 50 hourly bond returns during a window of 1 week, sampled from 08:00 to 17:00 CET. To transform the bond yield time series $y_{i}^{t}$ into a bond return time series $r_{i}^{t}$, we apply a duration approximation: $r_{i}^{t}\sim -D_{i}^{t}\left(y_{i}^{t}-y_{i}^{t-1}\right)$ with duration $D_{i}^{t}$ for bond *i* at time *t*.

To extract the correlation influence *d*_*i,j*:*k*_ from one market *k* to the correlations of another market *i* to all other markets *j*, we employ a definition of correlation influence *d*_*i,j*:*k*_ = *C*_*ij*_ − ρ_*ij*:*k*_ from Kenett et al. (2010) based on partial correlations $\rho_{ij:k}=\frac{C_{ij}-C_{ik}C_{kj}}{\sqrt{1-C_{ik}^{2}}\sqrt{1-C_{kj}^{2}}}.$ If the correlation influence is positive, the return time series of market *k* has a positive, converging influence on the correlations between the return time series of markets *i* and *j*. If the correlation influence is negative, the return time series of market *k* has a negative and diverging influence on the correlation between the returns of markets *i* and *j*. We average across market *j* to get the average correlation influence *d*_*i,k*_ = 〈*d*_*i,j*:*k*_〉_*j*≠*i,k*_. This asymmetric matrix reflects a directed graph from *k* to *i*.

To reduce the number of directed links in the resulting correlation influence network, we employ a bootstrap (Efron, 1979) filter that only retains the directed links *k* → *i* if and only if |*d*_*i,k*_| > *Q* × σ_*bootstrap*_(*d*_*i,k*_) with a parameter *Q* = 3. *Q* is not a convergence parameter, as it only filters out more links at a higher *Q*. We compute σ_*bootstrap*_(*d*_*i,k*_) with a resampling with the synchronous replacement of the cross-section of bond returns. Following Politis and Romano (1992), we draw the block length from a uniform distribution between 1 and 10 h for each sample to account for serial correlation.

This method does not involve a time lag between the time series of the respective markets and thus addresses only synchronous effects. In contrast to Beetsma et al. (2017) and Van Der Heijden et al. (2018), the news events themselves are not explicitly part of the model.

Partial correlations have also been employed by Saroyan and Popoyan (2017) to analyse risk spillover between European bank and sovereign credit risk. They find contagion from other countries to the correlations between the CDS spreads of banks and the sovereign bonds of their home country and recommend non-zero risk weights for sovereign bond holdings of banks.

Giudici and Parisi (2018) integrated partial correlation networks into a structural VAR model, labeled CoRisk approach. They find high contagion risk for peripheral countries from other peripheral countries, but low contagion risk between core and periphery. These findings confirm our results of a strong core-periphery segmentation, visible in the persistent block structure of the bond return correlation matrices.

To enable a more detailed discussion of this block structure, we analyse the blocks using a non-parametric clustering method. We apply a hierarchical clustering method (Ward, 1963) using the distance matrix metric $G_{ij}=\sqrt{2\left(1-C_{ij}\right)}\;$ as a function of the bond return correlation matrix *C*_*ij*_ according to Gower (1971). This choice of the distance metric preserves the sign of the correlation coefficients, which is important as we specifically want to discriminate positive from negative correlations. In contrast to the standard portfolio management literature, negative correlations are not an opportunity for diversification, but a warning signal in the specific case of this dataset as they appear between Euro area sovereign bonds that should be benchmark instruments without default risk.

To assess the quality of the hierarchical clustering compared to a simpler *k*-means clustering algorithm, we employ the “average silhouette width” criterion as suggested by Rousseeuw (1987). According to Rousseeuw, a higher number for the average silhouette width points to a more appropriate clustering. Table 1 shows a comparison of the average silhouette widths of the hierarchical and the *k*-means clustering for different values of *k*. For larger values of *k*, hierarchical clustering shows higher average silhouette widths. The null hypothesis of hierarchical clustering not leading to higher average silhouette widths than *k*-means clustering could be rejected with a *p*-value of 1% for the dataset given by the three discussed time periods and *k* values from 2 to 6.

**Table 1:** Average silhouette widths for hierarchical and k-means clustering.

***k***	**2**	**3**	**4**	**5**	**6**	**Avg**
Hierarchical	0.65	0.62	0.67	0.70	0.75	0.68
k-means	0.65	0.62	0.66	0.69	0.70	0.66

*The p-value of the t-test for the mean difference of the average silhouette width between the hierarchical clustering and k-means for each k is ≤ 1%*.

From the viewpoint of the specific application domain of European bonds, hierarchical clustering has the additional advantage of making overlapping correlation blocks visible. Following Gower and Ross (1969) and Mantegna (1999), we present the membership of the various bond markets to a hierarchy of clusters using a dendrogram. The clusters at the lowest levels of the dendrogram correspond to the most pronounced blocks in a correlation matrix. We found almost the same clusters using “complete linkage” or “single linkage” methods instead of Ward's method.

The advantage of a dendrogram compared to a heatmap is the objective representation of the clusters, as they are sorted in clusters according to the distance metric, whereas the visual impression of a correlation matrix as a heatmap depends on the predefined ordering. This ordering may depend on subjective beliefs or a market practice to sort issuers into a tiered hierarchy.

## Discussion

In the Discussion section, we discuss the bond return correlation matrices, hierarchical clusters and filtered correlation influence networks for the three political situations “Brexit referendum,” “French presidential elections,” and “Italian budget negotiations” as main results. A supplementary spreadsheet offers more technical details:

Supplementary Table 1 shows the correlation matrices as numbers.

Supplementary Table 2 shows the filtered average correlation influences as numbers.

Supplementary Table 3 shows investor flows in EFSF/ESM bonds.

Supplementary Figure 1 shows the results of *k*-means clustering with *k* = 4.

Supplementary Figure 2 shows silhouette widths for *k*-means and hierarchical clustering for different values of *k* to compare the performance of both clustering methods.

Supplementary Figure 3 shows the cumulative outgoing filtered correlation influences per market.

### Brexit Referendum

**Figure 2:**
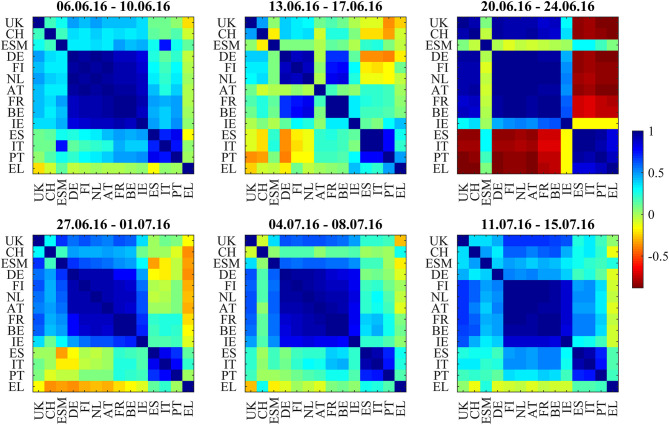
Bond return correlations during the weeks around the Brexit Referendum (23.6.2016).

**Figure 3:**
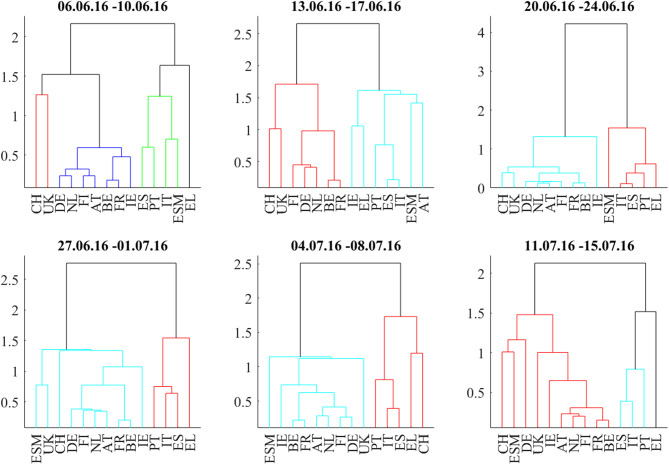
Dendrograms during the weeks around the Brexit Referendum (23.6.2016).

**Figure 4:**
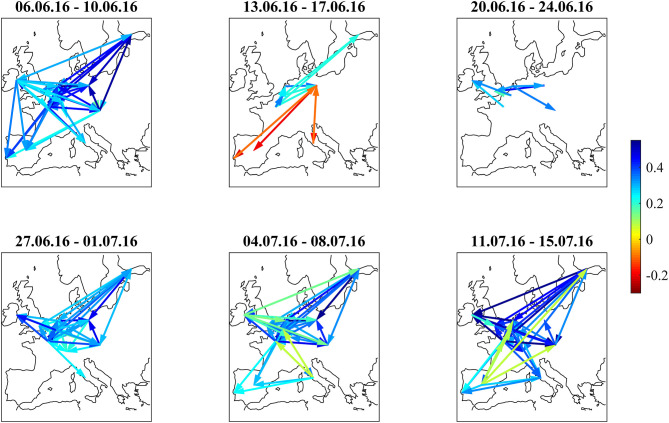
Filtered correlation influence networks during the weeks around the Brexit Referendum (23.6.2016).

**Figure 5:**
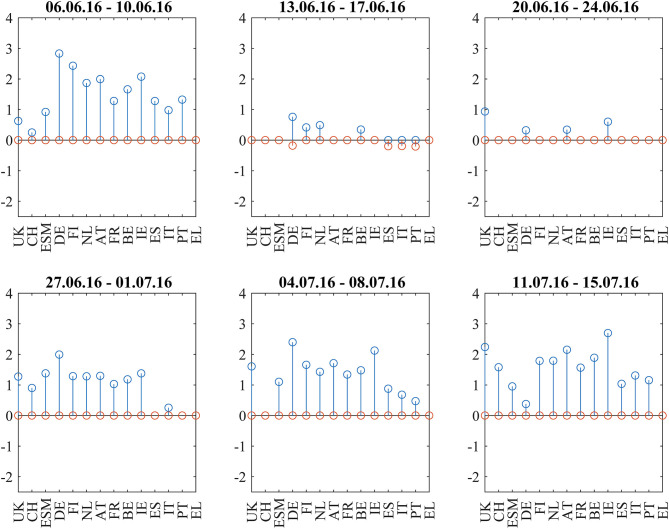
Cumulative positive (blue) and negative (red) incoming filtered correlation influences per market during the weeks around the Brexit Referendum (23.6.2016).

We discuss the first situation describing the weeks around the 2016 Brexit referendum using Figures 1B, 2–5: Figure 1B shows the odds of the “leave” outcome as estimated by the British bookmaker odds comparison service “Oddschecker” (Bloomberg ticker: ODCHLEAV Index) and the GBP exchange rate. In the weeks before the referendum, the odds for “leave” hovered in a range between 23 and 43%. The British pound exchange rate against the Euro inversely mirrored these odds. After the referendum, the odds massively underestimated the outcome and jumped from 23 to 100%, with the British pound losing almost 9% against the Euro in 2 days. Figure 2 shows the correlation matrix of hourly bond returns during the weeks before, during and after the referendum (June 23). Figure 3 shows the results of Wards' hierarchical clustering as dendrograms. Figure 4 presents the filtered correlation influence networks during the same weeks on geographical maps. Figure 5 shows the cumulative positive (blue) and negative (red) *incoming* filtered correlation influences per market. The *outgoing* filtered correlation influences are shown in Supplementary Figure 3.

Two weeks before (June 6–June 10) the referendum, the correlation matrix showed strong positively correlated core/semi-core and periphery blocks, and positive to neutral correlations between core/semi-core and periphery. UK bonds show weak positive correlations to the European core and semi-core. The core/semi-core block has only a very weak substructure. Irish bonds belong to the core/semi-core block. The dendrogram for this week confirms the block structure. The *k*-means clustering assigns a discrete cluster number from 1 to 4 to each of the bond markets but does not relate the four clusters to each other. The *k*-means cluster assignments are roughly consistent with the results from the hierarchical clusters but deliver a more “binary” view. For example, Italy belongs to the ESM cluster in both clusterings, but only the hierarchical clustering shows the tight coupling of Italy to Spain and Portugal one hierarchy level above. Throughout the 6 weeks with very few exceptions, we see, in the dendrograms, Greece, Portugal, Spain, and Italy as main constituents of the periphery block, Germany, Netherlands, Finland, and Austria as main “core” countries and Belgium, France, and Ireland as main “semi-core” countries.

Interestingly, UK stays very close to the core block, as well as Switzerland. ESM is also part of the core block except for the Brexit week where it was hierarchically part of the periphery. It moved back to the core a week later, after worries about the further European integration had quickly calmed down.

The correlation influence network shows strong connections within and between core and periphery.

During the week directly before (June 13–June 17) the referendum, the smaller issuers ESM, Austria, and Ireland decorrelate. Spain, Italy, and Portugal develop slightly negative correlations to Germany. Portugal also shows slightly negative correlations to British and Swiss bonds. The dendrogram for this week shows members leaving the clusters compared to the week before. The network (Figure 4, second panel) shows negative filtered correlation influences between Germany and the three peripheral countries Spain, Italy, and Portugal. These negative influences are statistically significant, as they pass the noise filter, but of small amplitude (Figure 5, second panel). Only a few core countries are affected by positive correlation influences.

The week of the referendum (June 20–June 24) induced strong positive correlations within the core and periphery blocks, and very strong negative correlations between core and periphery. UK and Swiss bonds were highly correlated to the “core Europe” block and thus also negatively correlated to the Euro area periphery (Spain, Italy, Portugal, Greece). The British currency absorbed the negative shock of the referendum to the UK economy. British bonds even gained in market value, consistent with the core Euro area bonds. The dendrogram of this week confirms the strong core-periphery segmentation. The network shows only a few connections that pass the noise filter.

During the 3 weeks after the referendum (June 27–July 15), correlations returned to the first week in the panel. Irish bonds return to the core/semi-core block. The first and the last week of the correlation matrix panel look very similar, also the dendrograms and networks.

From June 6 to July 1, the net flows from Asian investors into EFSF/ESM bonds were balanced. Two weeks and 3 weeks after the referendum (July 4–July 8 and July 11–July 15, net flows were negative at about −0.5 bn EUR, respectively. These flows after the referendum may be completely independent of the political event, or they may be a reversed flight-to-safety reaction (i.e., outflows from the safe haven when the political situation normalizes.

### French Presidential Elections

The second situation begins 3 weeks before the first round of the 2017 French presidential elections and ends 1 week after the second round. Figure 1C shows the odds of Le Pen winning from Oddschecker (Bloomberg: ODCHFRML Index) together with the spread of 10Y French bonds vs. 10Y German bonds. The spread decreases from 73 bp with the Le Pen odds until 50 bp at the first round (April 23, resulting in the second round between Le Pen and Macron) and then further until 43 bp at the second round (May 7, resulting in the victory of Macron).

**Figure 6:**
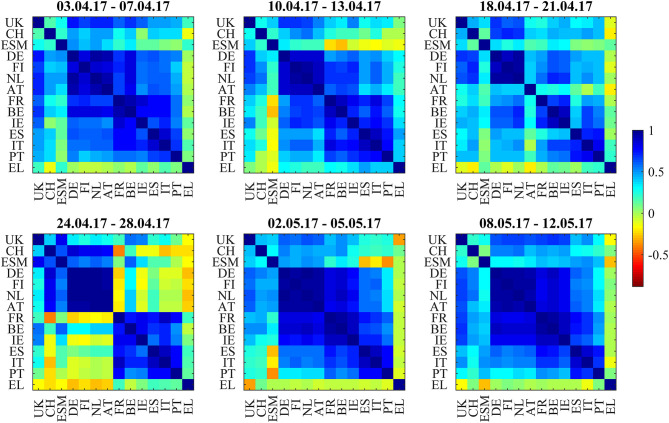
Bond return correlations during the weeks around the 2017 French Presidential Elections (first round: 23.4.2017, second round: 7.5.2017).

**Figure 7:**
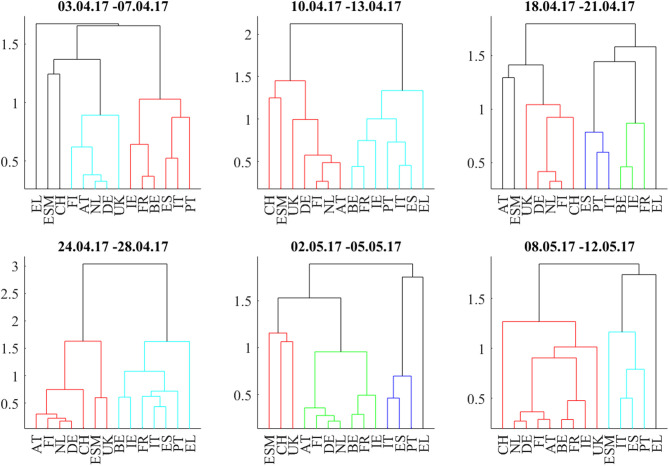
Dendrograms during the weeks around the 2017 French Presidential Elections (first round: 23.4.2017, second round: 7.5.2017).

**Figure 8:**
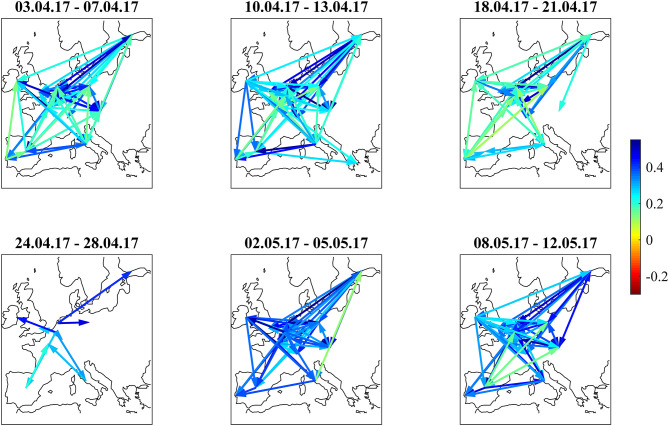
Filtered correlation influence networks during the weeks around the 2017 French Presidential Elections (first round: 23.4.2017, second round: 7.5.2017).

**Figure 9:**
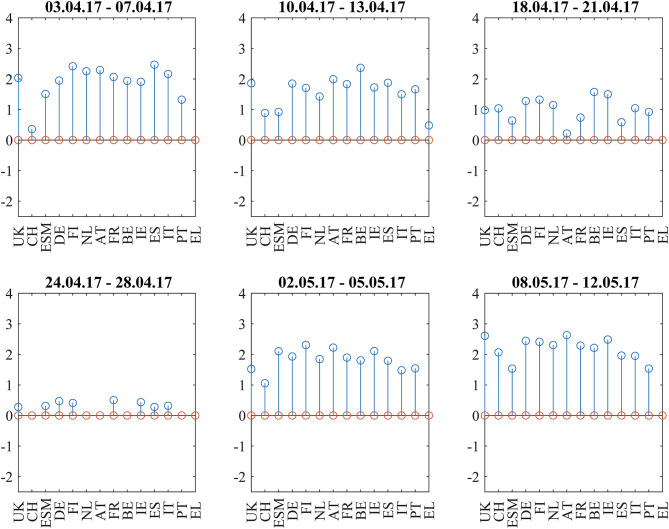
Cumulative positive (blue) and negative (red) incoming filtered correlation influences per market during the weeks around the 2017 French Presidential Elections (first round: 23.4.2017, second round: 7.5.2017).

Figure 6 shows the bond return correlations as heatmaps. As the political position of France within the EU was an important topic of the elections, the position of French bonds within the European tier structure was a trading topic. The market challenged the usual structure of a “core” block (DE, FI, NL, AT), a “semi-core” block (FR, BE, IE), and a “periphery” block (ES, IT, PT). Especially in the week immediately after the first round (April 24–28), France was part of a “semi-core plus periphery” block (FR, BE, IE, ES, IT, PT) and showed slightly negative correlations to Swiss bonds. After that, the block structure normalized. The dendrograms in Figure 7 confirm the “semi-core plus periphery” block in a corresponding hierarchy. A similar hierarchy is already visible in the second panel (April 10–April 13) of Figure 7. The dendrograms hence show that the uncertainty around France was affecting the “semi-core” block as a whole. Uncertainty stopped 1 week after the first round when the other candidates endorsed Macron such that it became likely that he would win the second round. The correlation influence networks in Figure 8 confirm the weakening of the established block structure until April 28 and recovery to an almost fully positively connected network afterwards. In contrast to the 2015 Greek negotiations and the 2016 Brexit referendum, there are no negative correlation influences during these 6 weeks (Figure 9).

The net flows of Asian investors into EFSF/ESM bonds are substantially positive (+384 mln EUR) in the week from April 3 to April 7 and in the week after the first round (+251 mln EUR from April 24 to April 28. The net selling in this week is most probably a technical flow: investors swap old bonds to the new issuance. Important is here the positive net volume, showing additional buying of the issued volume.). After that, they are negative during the weeks before and after the second round (−166 mln EUR from May 2 to May 5 and −133 mln EUR from May 8 to May 12). We interpret the data as a flight-to-safety movement with a reversal after the result from the second round: Asian investors were, in sight of a political event with an uncertain outcome, increasing their “core block” exposure (where the correlations clearly show that EFSF/ESM belong to) at the cost of peripheral bonds. Consistent with this interpretation, French bonds traded at a 30 bp risk premium to the yield of ESM bonds at the beginning of 2017. This spread decreased to zero at the end of the second quarter of 2017, as it did with respect to other core block bonds such as Bunds.

### Italian Budget Negotiations

Figure 1D shows the main observable of Italian fiscal and EU political discussions, the spread between Italian and German 10y bonds (IT-DE) from January to October 2018. At the beginning of the year, the spread was at 150 bp on par with the spread of Portuguese bonds (PT-DE) and about 50 bp higher than the spread of Spanish bonds (ES-DE). After the electoral success of Five Stars and Lega in early March, the Italian spread decorrelated from Portugal and Spain. As the new government was set up at the end of May, the spread widened by an additional 100 bp. During the negotiations within the new government about the budget given the electoral promises to increase spending and frequent postures against the EU budget rules, the spread showed increased volatility in several waves until October 19 when it reached 336 bp. Portuguese and Spanish bonds traded in much lower ranges, showing only mild contagion.

**Figure 10:**
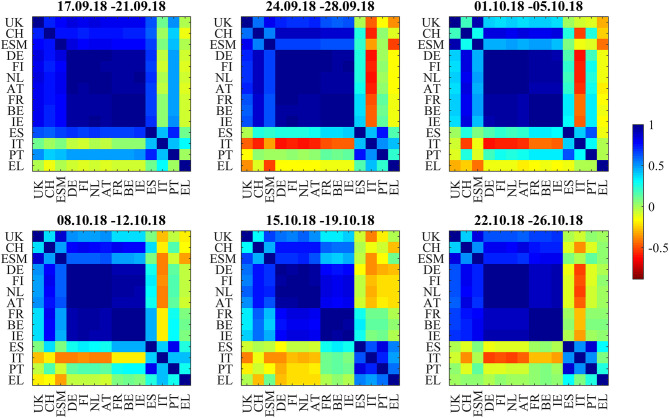
Bond return correlations during the Italian budget negotiations of autumn 2018.

**Figure 11:**
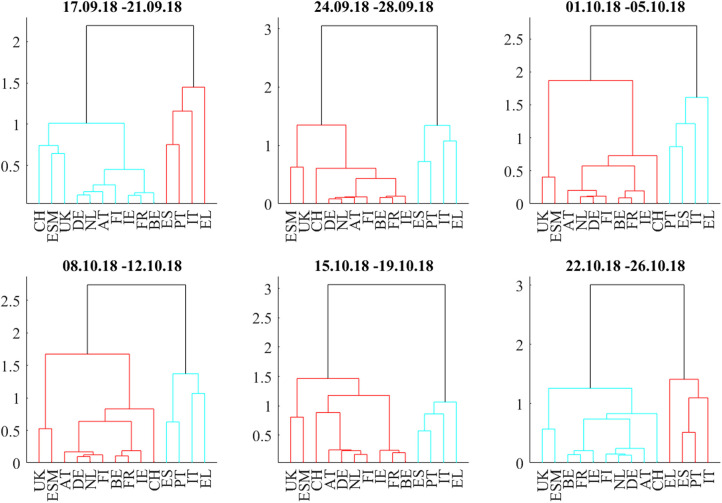
Dendrograms during the Italian budget negotiations of autumn 2018.

In Figure 10, the correlation heatmaps show positive correlations within and between the core and semi-core blocks and positive correlations to the ESM bonds and the non-Euro denominated UK and Swiss bonds throughout the full 6-week period from September 17 to October 26. The boundary between the core and semi-core block is barely visible but consistent. The correlations of the two peripheral countries, Spain and Portugal, to the semi-core countries are between neutral and strongly positive. The correlations between Italy and the core (AT, DE, FI, NL) and semi-core (BE, FR, IE) are between neutral and strongly negative. Greece (EL) decouples and sometimes shows negative correlations to ESM, CH, and UK bonds. The dendrograms in Figure 11 confirm the consistent core and semi-core blocks, the strong coupling between Spain and Portugal and the isolated role of Italian bonds until the third week. During the fourth week (October 8–12), Italy forms a cluster with Greece. In the fifth week (October 15–19), a periphery block with Spain, Italy, Portugal, and Greece is visible both in the correlation matrix and in the dendrogram. This block weakens in the last week (October 22–26). It is noteworthy that the block structure “core,” “semi-core,” and “periphery” remained constant through the observation period in the dendrograms. The intact block structure means that every yield movement on the Italian bond market affected the other peripheral markets more as markets belonging to the other blocks. In other words, while the level of correlation and influence changed within the observation period, the fundamental structure remained unchanged.

**Figure 12:**
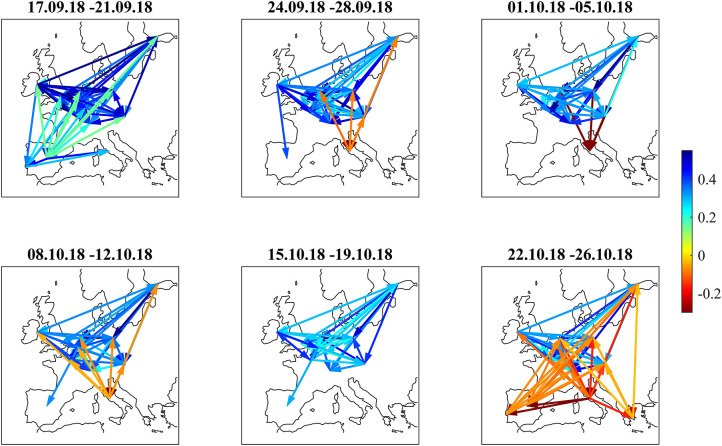
Filtered correlation influence networks during the Italian budget negotiations of autumn 2018.

The correlation influence graphs in Figure 12 show strong positive influences between the core and semi-core countries and toward Spain and Portugal in the first week, whereas Italian bonds couple positively to Spain. In the second week (September 24–28), all core countries develop negative correlation influences toward Italy. This sentiment improvement is confirmed in the third week (October1–5). During the fourth week (October 8–12), there are negative correlation influences between Italy and all core and semi-core countries. Spain recoupled to the semi-core in the fourth week. During the week from October 15 to October 19, positive correlation influences within core and semi-core bonds passed the noise filter. The budget submitted by the Italian government on October 16 was rejected on October 18 by the EU commission.

**Figure 13:**
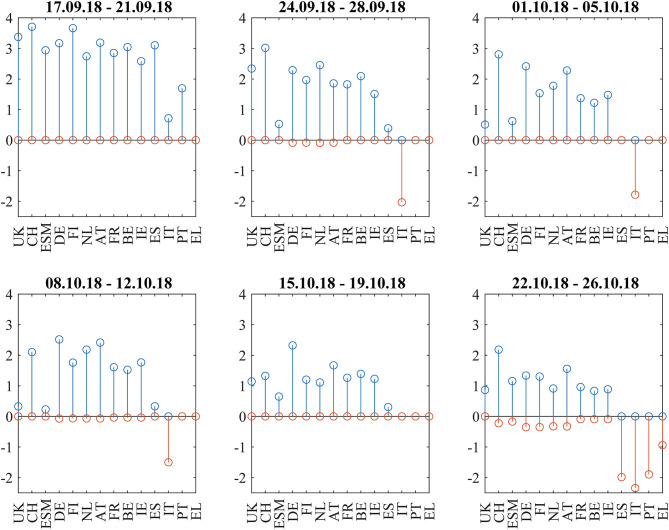
Cumulative positive (blue) and negative (red) incoming filtered correlation influences per market during the Italian budget negotiations of autumn 2018.

During the last week (October 22–26), Equities sold off as the EU commission formally requested the Italian government to revise their budget within 3 weeks. Negative correlation influences were visible between the core European block and all peripheral countries and from Italy to the rest of the periphery. The amplitudes of these negative correlation influences are larger (Figure 13) than during the Brexit referendum and French election cases. This pattern echoes the frequent spillover patterns during the 2015 negotiations between the Eurogroup and Greece (Schwendner et al., 2015).

The net flows of Asian investors into EFSF/ESM bonds were close to zero in the period from September 17 to October 5. In the week from October 8 to October 12, net selling of 187 mln EUR was overcompensating primary purchases of 136 mln EUR. In the week from October 15 to October 19, there was net buying of more than 1 bln EUR, more than 90% of it on the primary market. In the week after that, we saw only little net inflows of 91 mln EUR on the secondary market. These flows reflect the increasing buying from Asian investors in the fourth quarter of the year; still the volume in the time window of this case study was above average. On the background of the political scenery, the inflows may be attributed to steady investment in quality, if not even be interpreted as flight-to-safety, taking into account the above-average volume. Flight-to-safety movements usually happen at a higher pace than the reverse ones since risk protection usually has more urgency than the relaxation of risk protection measures. Also, there has not been any strong political signal letting investors move toward a “risk-off” mode. Hence, we do not see any reverse flight-to-safety in the observation period of 6 weeks.

## Conclusion

In an empirical study, we discussed the European bond market return correlations in three prominent events during 2016–2018. In contrast to the frequent spillover patterns that happened during the negotiation between the Eurogroup and Greece in 2015 (Schwendner et al., 2015) about the third financial assistance programme, the patterns around the 2016 Brexit referendum, the 2017 French presidential election and the 2018 budget negotiations in Italy were different.

The 2016 Brexit referendum only caused a muted warning signal in the form of negative correlation influences from German to Spanish, Italian, and Portuguese bonds in the week before the referendum and stronger core-periphery distortions with volatile correlations during the week of the referendum due to the unexpected result. The pattern of negative correlation sentiment reversed quickly. However, the devaluation of the British pound remained.

The 2017 French presidential elections showed a merge between the semi-core correlation block and the periphery correlation block before the second round, but no negative correlations or correlation influences between core and periphery. The French bond spreads improved after the second round.

Finally, the Italian budget negotiations in autumn 2018 showed increased spreads for Italian bonds and negative correlation influences between core Europe and Italy. During the last week from October 22 to 26, a significant pattern of negative correlation influences from core Europe and Italy to the rest of the periphery was visible.

Interpreting the primary and secondary market aggregated net flows of Asian investors in the context of euro area bond correlations, we observe an interesting relation: we saw flight-to-safety patterns into ESM bonds in the two case studies where ESM was, in terms of correlations, part of the core block. In contrast, during the week of the Brexit referendum, the ESM correlations did not show significant relations, and the flows did not show clear patterns. With the quick calming down of the markets, the normal core structure with ESM being part of it was visible again.

## Author Contributions

MS implemented the analytics. PS wrote the main parts of the paper and produced the figures. MH contributed to the discussion section. All authors are accountable for the content of the work together.

### Conflict of Interest

The authors declare that the research was conducted in the absence of any commercial or financial relationships that could be construed as a potential conflict of interest.
